# A randomised crossover trial of staff time with proned patients in the ICU using the ‘BathMat’

**DOI:** 10.1186/s13063-025-09221-x

**Published:** 2025-11-13

**Authors:** Jerome Condry, Andy Georgiou, Anders Vangsgaard, Harry Mills, Theresa Smith, Alistair Hunt, Alexander J. G. Lunt

**Affiliations:** 1 Royal United Hospitals Bath NHS Foundation Trust, Bath, BA1 3NG UK; 2 University of Bath, Claverton Down, Bath, BA2 7AY UK

## Abstract

**Background:**

The technique of turning a ventilated patient into the prone position is one which has a strong evidence base for improving both oxygenation and patient survival in the intensive care unit (ICU). As such, proning is advocated in national and international guidance. To mitigate the risk of complications, national guidance recommends repositioning proned patients every 2–4 h. The current process of repositioning is associated with risk and requires multiple staff members recurrently during the period of prone therapy. There is therefore an urgent need to explore safer and more efficient techniques. To address these issues, a multi-vessel inflatable pillow (the ‘BathMat’) has been developed which aims to improve the safety and ease of repositioning, whilst reducing the number of staff and the time required. Inflation of the BathMat, which is placed beneath a proned patient, allows easy repositioning of the head and arms, before deflation returning the patient to the resting position. The device requires clinical testing to assess its benefits outside of simulated scenarios.

**Methods:**

The BathMat trial is a randomised, multicentre, single-blind crossover study of repositioning in ventilated proned patients in ICU using the BathMat vs conventional care. Ventilated patients who require proning will be randomised to be repositioned with standard care or the BathMat. The same repositioning method will be used throughout each proning session, but will alternate on subsequent proning sessions between BathMat and standard care. The primary outcome is the difference in total staff time (number of staff × total time taken) required to reposition a patient with the BathMat compared with standard care. Secondary outcomes are the number of repositioning exercises performed, the number of adverse incidents associated with repositioning, the ease of manual handling, and the health economic costs of the complications of proning.

**Discussion:**

This is the first clinical trial of a device specifically designed to improve the quality of patient care delivered and improve the staffing resources required to care for proned ICU patients. The impact potential for patients and staff is significant, through liberation of staff, improved manual handling, and risk mitigation for proned patients.

Trial registration

ClinicalTrials.gov NCT06844617. Registered on Jan. 19, 2025. https://clinicaltrials.gov/study/NCT06844617

**Supplementary Information:**

The online version contains supplementary material available at 10.1186/s13063-025-09221-x.

## Administrative information

Note: the numbers in curly brackets in this protocol refer to SPIRIT checklist item numbers. The order of the items has been modified to group similar items (see http://www.equator-network.org/reporting-guidelines/spirit-2013-statement-defining-standard-protocol-items-for-clinical-trials/).

**Table:** 

Title	The ‘BathMat’ Trial A randomised crossover trial of staff time with proned patients in the ICU using the ‘BathMat’
Trial registration	Protocol version E (Jan 2025) ClinicalTrials.gov reference NCT06844617
Funding and support in kind	Funder(s) National Institute for Health Research (NIHR) Grange House, 15 Church Street, Twickenham, TW1 3NL Financial and non-financial support given This work was supported by the National Institute for Health Research Invention for Innovation (I4I) Product Development Award (Ref. NIHR206410)
Authors	Dr Jerome Condry^1^, Dr Andy Georgiou^1^, Anders Vangsgaard^2^, Harry Mills^2^, Dr Theresa Smith^2^, Dr Alistair Hunt^2^ and Dr Alexander J G Lunt*^2^ 1. Royal United Hospitals Bath NHS Foundation Trust, Bath, BA1 3NG, UK 2. University of Bath, Claverton Down, Bath, BA2 7AY, UK *Corresponding author email: ajgl20@bath.ac.uk
Trial sponsor contact	Jane Carter, Royal United Hospitals Bath NHS Foundation Trust, jane.carter14@nhs.net
Role of sponsor	This is an investigator initiated clinical trial sponsored by Royal United Hospitals (RUH) National Health Service (NHS) Foundation Trust. The sponsor holds overall responsibility for initiation and management of the trial but has no involvement in the study design; data collection, management, analysis, or interpretation; manuscript writing; or the decision to submit the report for publication. The research was funded by the NIHR, which likewise had no role in any of these aspects and will not exert ultimate authority over the conduct, analysis, or dissemination of this study

## Background and rationale

Approximately 235,000 patients per year are admitted to ICU in the UK. One of the most common reasons to be admitted to an ICU is the need for respiratory support. Evidence suggests that over 16,500 ICU patients per year (7%) [1] have lung injury of significant severity that proning will be beneficial to improve both oxygenation and chances of survival. Proning describes the technique whereby sedated patients on a ventilator are turned onto their front. Once proned, patients stay on their front for 16–18 h. Depending upon the patient’s condition, this process may be repeated up to five times during an ICU stay, although during the COVID-19 pandemic, some patients were proned up to 10 times. The use of proning has been shown to reduce the risk of death by 17.4% [2]; this is one of the greatest reductions in mortality from a single intervention in intensive care medicine, and this underpins the enthusiasm of clinicians to employ the technique. Whilst proned, a patient’s head needs to be turned and their arms repositioned (hereafter termed ‘repositioning’) regularly to minimise the risk of pressure sores and nerve/organ injuries. This task requires a team of five or more staff and takes 30–60 min to perform (more if full personal protective equipment (PPE) is required).

The evidence that proning improves oxygen levels and survival rates in patients who are severely oxygen dependent in ICU is irrefutable. As such, the technique forms an essential component of national and international guidance for ICU clinicians. Complications associated with proning are well described [3]. To mitigate the risk of complications, national guidance recommends repositioning every 2–4 h. Greater awareness of the benefits of proning following the COVID-19 pandemic means that the use of the technique may be more readily employed in the post-pandemic era. This increase, in combination with the unwieldy, demanding, and far from risk-free repositioning process (described below), means there is an urgent need to explore safer and more efficient repositioning techniques.

Repositioning is currently typically carried out in one of four ways:

1. The patient is slid up the bed to allow their supported head (now clear of the mattress) to be rotated before returning to the original position. This is the most common method in the UK [4] and is recommended in national and international guidance. It requires at least 5 staff and is associated with a significant manual handling exercise.
2. A hoist can be used to lift the patient off the bed to allow repositioning whilst elevated. Hoists are large devices that require 3–5 staff to operate, they can be difficult to manoeuvre and are typically limited in number on an ICU. This approach requires the insertion/removal of slings, takes approximately the same amount of time as a manual turn described above, and can lead to unpredictable movement of the patient whilst suspended.
3. A specialist proning bed can be used. This is prohibitively expensive (£800+ per day), complicated, and requires large areas for storage when not in use. This approach requires a specialist bed per patient, thereby limiting the number of patients that can be simultaneously proned on any given ICU. This approach is the least preferred and least used repositioning method.
4. Patients can be lifted by staff on the sheet on which they lie whilst one individual turns the head. This requires at least 5 members of staff and is exceptionally demanding from a manual handling perspective, particularly if patients are obese.

Repositioning patients is a physically demanding and time-consuming task for ICU staff, but it is vital for patient care. The high workload involved places a significant strain on staff capacity and contributes to several risks:

i. At least 5 staff are required to reposition the patient every 2–4 h. This mandates drawing staff from across the ICU and leads to nurses having to leave other patients to support the repositioning exercise. This results in recurrent interruptions in care for other patients who are critically dependent on the drugs or machines which the nurses oversee. This practice necessitates breaches of national standards on nurse:patient ratios and jeopardises the lives of other patients.
ii. Any adverse event of an unsupervised patient on ICU would be all but impossible to defend medico-legally; liberating nurses to care for their own patients is therefore vital.
iii. Staff shortages on ICU in the post-pandemic era may be more significant than they were pre-pandemic, exacerbating the challenges of liberating staff to perform repositioning.
iv. The process of repositioning is currently far from risk free. As sedated, ventilated patients are moved about the bed, there is a risk of removal or displacement of lines or tubes on which the patient’s life is continually and critically dependent. This occurs in up to 12.5% of proned patients [3].
v. Improper repositioning risks damage to organs such as the eyes, liver, pancreas, or genitals and exposes patients to pressure sores, which are the most common source of litigation of ICUs in the UK [5].
vi. Recurrent repositioning is manually demanding for staff. Manual handling injuries cost the NHS £18bn in 2019/2020 [6], and any device which reduces this risk will ease staffing problems and the fiscal burden on the NHS as a whole.
vii. Staff burnout remains an ongoing challenge on ICU [7], deepening the staffing crisis. A device which unburdens staff will reduce burnout and staffing challenges.

This has led to the development of the inflatable prone repositioning device (IPRD) known as the ‘BathMat’ that is intended to improve the safety and ease of repositioning patients whilst reducing the number of staff and the time required. The BathMat consists of a multi-vessel inflatable pillow, placed underneath a proned patient, which can be inflated to raise the patient’s chest and hips. With the patient raised, repositioning of the head and arms can occur with ease, before the device is deflated and the patient is returned to the resting position. The BathMat has four main benefits for patients and staff:

1. Repositioning of the head and arms occurs without the risks described above.
2. It reduces the number of staff required from 5 to 2.
3. It reduces the time it takes for repositioning from 30–60 min to 10 min.
4. It removes the majority of the manual handling workload.

This study is required to demonstrate the effectiveness of this device in the clinical setting and provide data regarding its safety, usability, and reliability.

## Study design, aims, and objectives

### Aim

To determine the change in total staff time required for repositioning proned patients using the BathMat and to assess the safety of use when compared with standard care.

### Objectives and outcomes

See Table 1.

**Table 1:** Objectives and outcome measures

Objective	Outcome measure	Source
Primary objective To investigate the staff time required to perform a proned patient reposition exercise. This will include the total number of staff required as well as the duration of the exercise	a. Difference in number of staff required to reposition each patient b. Difference in time required to reposition each patient	The number of staff required to reposition the patient and the time required will be collected by clinical teams by scanning a quick response (QR) code unique to each patient every time a patient is repositioned
Secondary objectives a. Assessment of the number of repositioning exercises undertaken for each patient within a proning intervention b. The number of minor or major adverse incidents associated with repositioning patients whilst in the prone position. This includes events such as line or tube displacement c. The manual handling requirement to reposition d. In patients who suffered a major or minor adverse event (as defined in secondary outcome b), assessment of the healthcare costs relating to that event at 3 months following ICU discharge	a. Number of documented repositioning exercises b. Adverse events documented c. Difference in Likert scale measure of difficulty of repositioning between control and intervention groups d. Healthcare costs of adverse event at 3 months post discharge	a. Clinician form entry from QR code (as above) b. Clinician form entry from QR code (as above), research team form entry c. Clinician form entry (from QR code as above) d. Research team form entry

### Study design

**Fig. 1:**
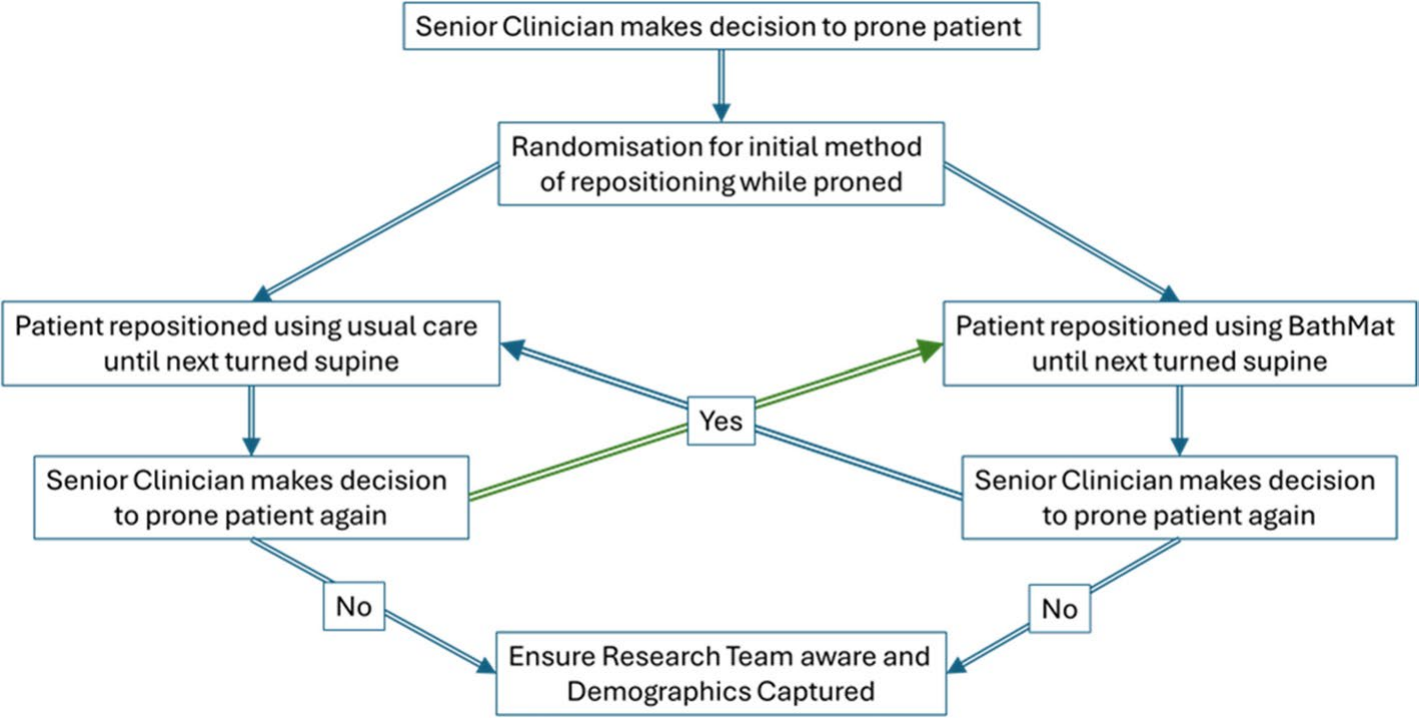
Study flowchart

This is a randomised, multicentre, single-blind crossover superiority study of repositioning in ventilated proned patients in ICU using the BathMat vs conventional care (Fig. 1).

#### Study setting

The trial will be performed across at least four adult ICUs within England. This is the minimum required to ensure adequate recruitment of 30 patients within the timescale of the trial and demonstrate external validity through recruitment across centres with a range of experience in proning. The use of multiple centres also facilitates recruitment of adequate numbers of patients within the 14-month trial and allows for comparison between differences in repositioning practice, allowing generalisability of the data acquired.

#### Sample size

A sample size of 30 patients was determined from statistical analysis of the proposed trial. This is sufficient to detect a decrease in repositioning time of at least 10 min with a power of over 95%. An estimated 10-min improvement was identified via a preliminary unpublished volunteer study. This was determined to be the optimal route to estimate the approximate magnitude of time saving before the device was trialled on patients as part of the proposed clinical study.

Power calculations were carried out using Monte Carlo sampling to allow for variability in the number of repositionings per proned session and the number of proned sessions per patient. In addition to standard deviation assumptions, 2–4 repositionings per proning session were used (randomly sampled with proportions (1/5, 3/10, 1/2)) and a dropout rate of 1/3 after the first session. The repeated measurements for each patient were simulated using a correlation of 0.7 within the same treatment and 0.3 between the two treatments.

#### Blinding and randomisation

Due to the nature of the device being tested, it is only possible to perform a single-blind study to evaluate the device. Participants will be randomly assigned to be repositioned on day 1 either with BathMat or standard care with a 1:1 allocation. Randomisation will be performed using SealedEnvelope.com with a block randomisation tool stratifying by site. Block sizes will not be disclosed to ensure concealment.

Clinical staff will enrol participants and assign intervention on recruitment by using SealedEnvelope.com, either online or via text service. Only participants meeting all inclusion/exclusion criteria will be allocated to an intervention.

#### Inclusion criteria

Any sedated, ventilated patient over 18 years of age identified as requiring proning by a senior ICU clinician.

#### Exclusion criteria

Patients who are:

- Awake
- Pregnant
- Under guardianship
- In their first proning session in the current ICU admission who have already been repositioned 2 or more times using standard care prior to recruitment to the study
- Over 200 kg
- Under 150 cm and over 205 cm
- Patients who have already been proned using conventional methods in the current ICU admission
- Patients who have broken skin on the anterior chest or abdominal wall

#### Withdrawal criteria

Participants will be withdrawn from the study if they do not supply or withdraw their consent for participation, or if their personal or nominated consultee opinion is that they would not wish to be included in the study.

## Device details

**Fig. 2:**
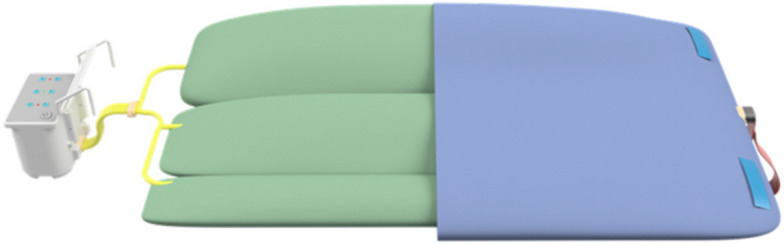
BathMat with cutaway section

**Fig. 3:**
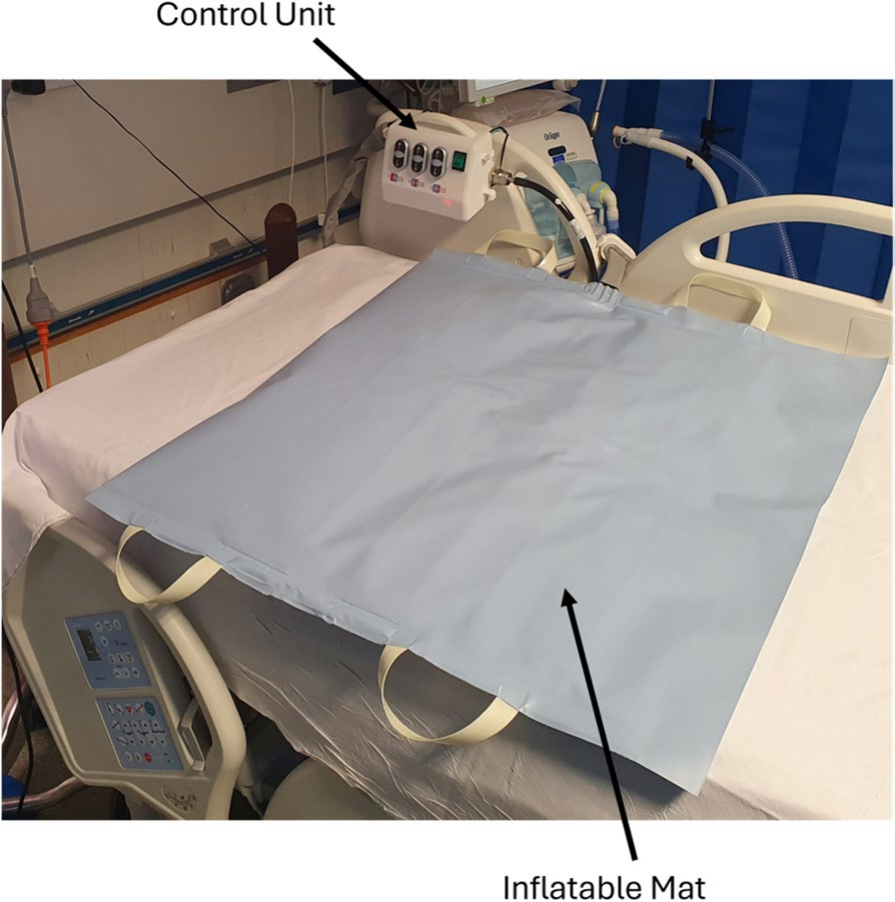
BathMat

The device, shown in Figs. 2 and 3, consists of an inflatable mat for single patient use and one reusable control unit. The functionality of the control unit will be assured by the trial team at the University of Bath. There are no special storage conditions for the device.

### Inflatable mat

The inflatable mat, consisting of three inflatable vessels of different sizes, is pictured in Fig. 2. It is designed to be placed between the mattress and the bedsheet, underneath the patient. Direct contact between the mat and any part of the patient should be avoided. The marked upper side will have a shoulder line to indicate appropriate positioning of the superior aspect of the patient’s shoulders. A non-interchangeable connector allows connection between the mat and the control unit.

### Control unit

**Fig. 4:**
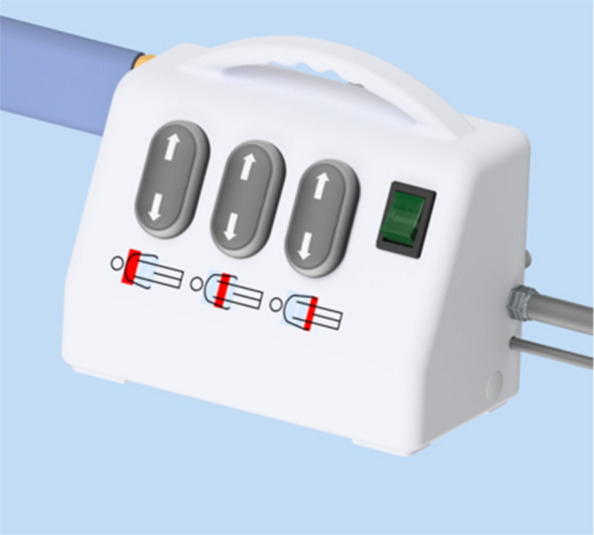
Control unit

The control unit (Fig. 4) is used to manually control the flow of air to and from the inflatable vessel, allowing a user to inflate and deflate the vessels as required in a bespoke fashion. It has three connections: an electrical power cable for connection to a wall socket, standard medical air hosing to connect to a 4 bar medical air outlet, and bespoke hosing to connect the control device to the inflatable vessel when required. The casing has an integral carry handle and hooks for tidying of cables for storage.

**Fig. 5:**
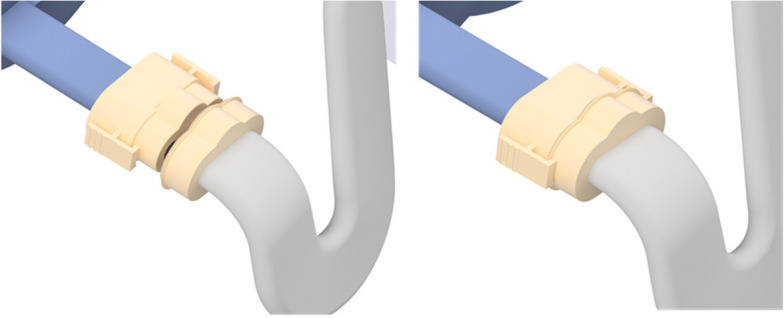
Bespoke connector

The bespoke hosing, arising from the left lateral aspect of the unit, is long enough to connect to the inflatable vessel with the unit hung from the side of the patient’s bed. A non-interchangeable connector (Fig. 5) only allows connection to the appropriate ports. Clasps either side of the connector provide a secure connection between the two aspects of the device. Gentle pressure releases these clasps and allows separation of the control unit and inflatable vessel when desired. In the unlikely event of connection problems between the control unit and the mat, each trial centre will be supplied with a spare control unit and replacement hosing.

The power switch is located on the left lateral wall of the control unit, marked by the universal power symbol. A power light indicates when the unit is switched on. If switched off, the control unit will not function, and no inflation is possible. There is no backup internal power source. In case of external power failure whilst inflated, the BathMat should be disconnected from the control unit to allow it to slowly deflate.

On the slanted superior aspect of the unit are the buttons to control inflation and deflation. Clearly marked to highlight which segment of the vessel they relate to, pressing each up arrow allows inflation of the relevant vessel. Pressing the down button results in deflation. If no buttons are pressed, the inflatable vessel will remain at the set inflation level.

## Training in use of device

All staff expected to be involved in repositioning will receive training in the use of the device in line with the instructions for use. This may be delivered by local or central study staff. Clinical staff will be responsible for the appropriate use of the device. Before using the device clinically, they may refresh training using a centrally produced training video or reviewing the training materials as required to ensure adherence to the instructions for use, which are also kept with the device controller at all times, with a quick reference guide should this be required by clinical staff.

Any staff receiving training in the use of the device will also receive training in the data entry requirements for assessing the primary outcome and other data only able to be recorded contemporaneously by clinical staff detailed in Tables 2 and 3. Any clinical staff using the device is required to have this training in order to use the device.

**Table 2:** Data to be entered by clinical team after each repositioning exercise

Repositioning start time Repositioning ready time Repositioning stop time Number of staff taken to reposition Cause for any delays in repositioning Complications during repositioning Perceived ease of repositioning Perceived acceptance of use of BathMat (if used) Other comments, reasons for delay in repositioning, etc.

**Table 3:** Repositioning complication domains

Tracheal tube blockage/displacement/extubation Infusion line kink/displacement Nasogastric tube displacement Bleeding from airway Other repositioning complication	

A training log will be kept by local site teams detailing staff trained and will be monitored centrally. Recruitment at each site will not begin until an adequate proportion of local clinical staff has received this training in order to ensure safe use of the device and appropriate data entry by clinical staff.

## Study procedures

**Fig. 6:**
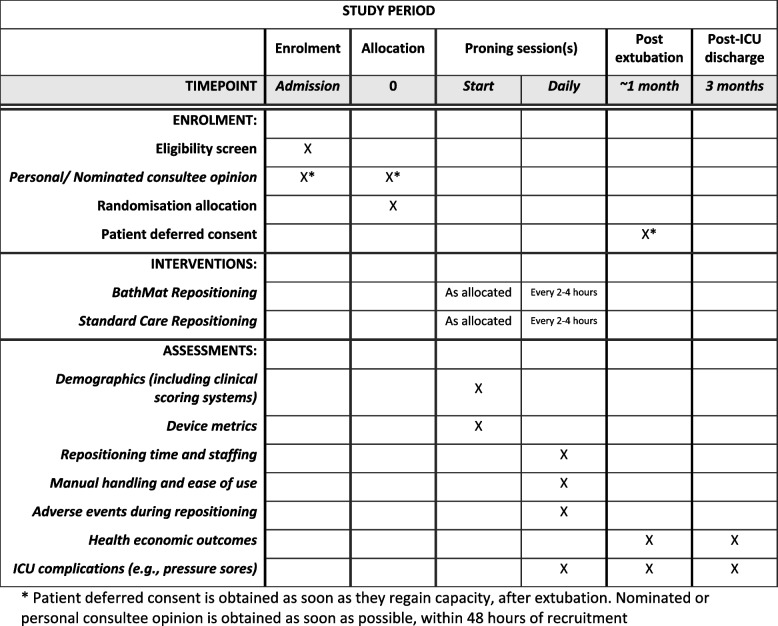
Schedule of enrolment, interventions, and assessments—BathMat trial

Patients ventilated and identified as suitable for proning by the ICU consultant/senior doctor will be randomised to be repositioned whilst in the prone position with either standard care or the BathMat on a 1:1 basis. The same repositioning method will be used throughout each proning session but will alternate on subsequent proning sessions between BathMat and standard care, if proned more than once. This will account for patient-to-patient heterogeneity in individual variables such as the requirement for PPE and body mass index. A total of 30 participants will be recruited, and each participant will be involved in the study. The SPIRIT figure for the trial is shown in Fig. 6.

### Recruitment strategies

Sites will be selected based on demonstrated capacity to recruit eligible participants, with feasibility assessments undertaken using retrospective audit data to confirm adequate case volume. Each site will maintain a prospective screening log capturing numbers screened, reasons for exclusion, and consent outcomes. These data will be reviewed regularly to monitor progress and identify any emerging barriers to recruitment.

To support consistent enrolment across sites, structured training will be provided to all staff involved in screening and consent. This will cover eligibility criteria, consent procedures, and communication techniques to ensure a clear and consistent approach. Recruitment materials, including posters and participant information leaflets, will be displayed in relevant clinical areas and designed to support understanding of the study and facilitate informed decision-making.

Performance will be reviewed through scheduled recruitment updates and communication between sites. Sites falling below expected recruitment trajectories will receive targeted support, which may include additional training or adjustments to local procedures. The recruitment target has been set based on conservative estimates and accounts for anticipated screen failures and non-consent. A multicentre approach ensures broader access to the eligible population and provides resilience should recruitment at any single site be lower than projected.

### Decision to prone and randomisation

Patients may be recruited to the study once a senior ICU clinician has made the decision to prone them. Any staff member trained to enrol patients may enact enrolment at any time of day or night by ensuring the patient conforms to the inclusion/exclusion criteria, recording patient details in the participant recruitment and randomisation form, and receiving the randomisation details. The recruitment and randomisation form will be accessed via QR code or hyperlink by the trained member of staff, and SealedEnvelope.com will be used for randomisation. Instructions on the form will then direct the randomising staff member to study documents located within their department. If patients are randomised to join the control arm, their first proning session will be carried out in accordance with usual proning and repositioning practices in the recruiting centre. If the patient is randomised to the intervention arm, the BathMat will be placed under the patient during the proning process and will be used for repositioning alongside all other standard care. Randomisation and recruitment to the study can be carried out before or after the patient has been proned in their initial proning session, as long as the patient has not undergone 2 or more repositioning exercises during their initial proning session before recruitment.

Participants will only be identified for recruitment by the direct care team, with any research team then subsequently assisting with the recruitment process as required.

Each proning session is expected to last 16 h in line with Intensive Care Society guidelines, with one proning session per day. There is no maximum or minimum number of repositioning exercises required per proning session, but any period of less than 2 h or more than 4 h between repositioning exercises (i.e. any frequency outside of national guidelines) will be investigated.

### Where patients are proned more than once

If a senior ICU clinician decides that more than one episode of proning is clinically indicated, the method of repositioning will alternate during each subsequent proning session as illustrated below. This illustration is intended for explanation only, and it should be highlighted that the number of times participants in the study are proned will be based solely on clinical indication and decision-making.

If proning is re-employed later in the patient’s ICU stay, the process of alternating the repositioning system will resume. For example, if a patient is proned on day 1 of their ICU admission and is randomised to standard care, and next requires proning on day 5 of the ICU stay, they will be repositioned using the BathMat on day 5, as per the table below.

**Table:** 

	1 st proning session	2nd proning session (if clinically indicated)	3rd proning session (if clinically indicated)	4th proning session (if clinically indicated)	5th proning session (if clinically indicated)
Randomised to standard care for repositioning	Standard care for repositioning	BathMat for repositioning	Standard care for repositioning	BathMat for repositioning	Standard care for repositioning
Randomised to BathMat for repositioning	BathMat for repositioning	Standard care for repositioning	BathMat for repositioning	Standard care for repositioning	BathMat for repositioning

### Placement of inflatable device

When a proned patient is due to be repositioned using the BathMat, the device will be placed underneath the patient and underneath their sheet either before or after they are proned, but before any repositioning exercise is planned. Participants will be turned prone in accordance with local ICU protocols. The placement of the BathMat will be checked to ensure the shoulder line on the BathMat aligns with the upper edge of the patient’s shoulders, the inflatable device has not wrinkled underneath the patient, and that the device and patient are positioned correctly on the bed. Ultimately, the final position of the patient on the mat must be decided by the clinical team, taking into account the size and shape of the patient but also the size and range of movement of the neck.

### Connection to control unit

Once the BathMat is appropriately positioned, the control unit is attached via the non-interchangeable connector. The clasps on either side of the connector should be checked to ensure they have adequately sealed the inflatable mat to the control unit.

### Inflation for repositioning with device

When a patient in the intervention (BathMat) group requires repositioning, the member of staff directly caring for the patient will contact relevant team members to assist with repositioning and prepare the patient. For repositioning with the device, a total of two members of staff are expected to be required. If more are used, the reason for this will be documented on the online form by the clinical team.

Once the patient is prepared, one suitably trained member of staff will control the patient’s head and airway, with a second team member using the control device. Inflation of individual vessels is controlled by the buttons on the control device, as seen in Fig. 4. Upwards arrows inflate the vessels, and downwards arrows deflate the vessels. Inflation is complete when the team assess the patient to be adequately lifted to allow repositioning. The maximum pressure that can be achieved in the vessel corresponds to the input pressure, and the vessel will stop inflating if this maximum pressure is achieved. Following rotation of the head, the device is fully deflated. The patient’s arms are moved when the patient has been returned to rest on the bed. Arm positioning is determined by standard unit protocols. The BathMat must always be left fully deflated in between repositioning sessions.

Repositioning in both groups must be performed every 2–4 h as per Intensive Care Society guidelines. At each point that this frequency is not achieved, the reasoning will be documented by the clinical team using the online data entry form, and will be recorded by the research team.

### Removal of device from patient

Once the proning session is completed, the patient will be returned supine as per standard ICU protocol. The inflatable device can be removed immediately before or after returning supine. First, the inflatable device is disconnected from the control unit by squeezing the clasps of the non-interchangeable connector and pulling the two halves apart. The patient can then be rolled to enable the device to be removed.

### Repositioning during standard care

Repositioning for patients during standard care will be performed as per local ICU protocol, every 2–4 h as per Intensive Care Society guidelines. If this frequency is not achieved, the reasoning will be documented in the clinician data entry form.

### Data entry by clinical team

When enrolled patients require repositioning, the total staff time taken to reposition must be recorded—this is the primary outcome measure. An online clinical data entry Microsoft form, accessible via QR code or hyperlink at the patient’s bedside using any device, will allow staff to submit data from Table 2. Table 3 details domains for repositioning complication. The live Microsoft form will collect patient data linked to the participant’s study number and be managed by the central study team. It will not contain any other identifiable information.

This requires the bedside nurse primarily responsible for patient care to record the time (HH:MM 24-h clock format) at each point of repositioning as stated below, and the number of staff required to do so, by scanning the ‘repositioning QR code for bedside nurse’ unique to that patient or using the appropriate unique uniform resource locator (URL). Time should be rounded to the nearest minute, without the use of decimal places or seconds. The form must be completed after each repositioning manoeuvre and does not require any timing calculations by clinical staff. Instead, the form asks for four data points from the bedside nurse regarding the primary outcome:

1. Repositioning start time: The time when the first staff member required to reposition the patient (in addition to the bedside nurse) enters the bed space to begin repositioning.
2. Repositioning ready time: The time when the last member of staff required to reposition the patient has arrived in the patient’s room or bedspace.
3. Repositioning stop time: The time when the last member of staff who was drawn in to assist the bedside nurse with repositioning has left the patients room or bedspace.
4. The number of staff taken to reposition: Includes all staff (including the bedside nurse) who have helped in the repositioning manoeuvre. This does not include anyone outside of the patient’s room or bedspace who was available for help, and not actively involved in the repositioning manoeuvre.

It should be noted that statistical analysis will be performed on differences in the time required to gather staff (the difference between repositioning start time and repositioning ready time) and the time required to perform the repositioning exercise (the difference between the repositioning ready time and the repositioning stop time). However, the overall repositioning time (the difference between repositioning start time and repositioning stop time) will be used in the calculation of the primary endpoint.

One member of clinical staff will be expected to complete this data entry exercise every time a patient is repositioned (with or without the BathMat). This is expected to be the bedside nurse looking after the patient, but may be contemporaneously delegated by them to any suitably trained individual.

### Data collection by research team

A representative from the research team will review participants in the trial whilst the participant is in ICU throughout the period they are being proned, or the first working day afterwards if this has occurred over the weekend; once they have regained capacity; and at 3 months post discharge. They will collect patient data as detailed in Tables 4, 5 and 6 including standardised risk scoring measures used in ICU with potential relevance to our study (Waterlow, SOFA scores) [8, 9]. Data on device metrics will be obtained through discussion with clinical staff caring for the proned patient. They will ensure complete data sets are recorded through access to the clinical data entry form and spreadsheet, collect personal and nominated consultee opinions as required, and obtain patient consent. Patient notes will not be accessed by members of staff outside of those in the direct team until consent has been obtained.

**Table 4:** Data to be collected by local research team—demographics

Patient metrics: Age, height, weight Frailty score Sequential Organ Failure Assessment (SOFA) [9] score on day 1 of proning Waterlow Pressure Ulcer Risk Assessment Scale on day 1 of proning [8] Pre-existing complications before day 1 of proning—see Table 6 ICU pressure sore risk score Medical comorbidities Diagnosis Device metrics—see Table 5 Proning complications—see Table 6

**Table 5:** Data to be collected by local research team—device metrics (where clinical team who have used device are available)

Ease of introduction under the patient Ease of inflation/deflation Suitability of speed of inflation/deflation Ease of repositioning—head/arms Manual handling issue/concern Other comments

**Table 6:** Data to be collected by local research team—proning complications

Pressure sore (face/chest/pelvis/knees/feet) Nerve injury (neck/arm/abdomen/leg) Blood vessel injury Skin abrasion Organ injury (liver/pancreas/genitals/ocular) Other	

The health economics questionnaire (309-08-19 (A) Health Economics Follow Up) must be completed for each patient at 1 month and 3 months after they have been recruited. This will be performed by a suitable member of the research team. If this form is not able to be fully completed by review of patient notes, the research team may need to contact the patient. This interview may be conducted in person if they are still an inpatient, or via telephone if they have been discharged. If contacting the patient by telephone, the research member will check the participant’s Electronic Staff Record (ESR) to ensure this remains appropriate.

### End of study

Participants end their involvement with the study when they no longer require proning and the study has a complete dataset.

### Consent

Patients become eligible for this trial during a period of critical illness. Proning is indicated in patients with moderate to severe lung injury and hypoxaemia. Prior to proning being considered, patients will have been sedated and ventilated as a life-saving measure during an emergency clinical situation. It necessitates the use of sedative and analgesic drugs (as part of standard care), leading to patients lacking mental capacity and/or the ability to communicate effectively. Moreover, the emergency clinical situation can cause profound distress for relatives, raising ethical concerns both about the burden of trying to understand the trial and the ability of a personal consultee (i.e. relative or close friend) to provide an opinion about trial participation during a time of great distress. For these reasons, attempts to obtain either prior informed consent from the patient or the prior opinion of a personal consultee are inappropriate.

We will adopt a Research Without Prior Consent (RWPC) model (also referred to as ‘deferred consent’), whereby eligible patients will be randomised to receive the assigned treatment. This is an accepted consent model in adult emergency and critical care research where participants lack mental capacity, and minimises the distress and additional burden on families during a distressing time. In addition, the urgent nature of treatments delivered in ICU means that any delay to commencing treatment could be detrimental to the patient (and to the scientific validity of the trial). This consent model is covered by an Emergency Waiver of Consent under the Mental Capacity Act (Research Ethics Committee (REC) review approved).

#### Patient informed deferred consent

Following randomisation, patients will be informed of their involvement in a research study once they are deemed to have regained capacity. A delegated member of the site research team will then approach them to obtain informed deferred consent. It is anticipated that this first approach will occur within 24–48 h of regaining capacity but should be performed as soon as appropriate: this may be prior to recruitment in some circumstances. Verbal consent for this discussion will be obtained by a member of the direct clinical team prior to this approach. A participant information sheet (PIS) will be given to the patient. The PIS will provide information about the background/rationale for the trial, what participation means for the patient (e.g. data collection, follow-up questionnaires), confidentiality and data protection, and the future availability of the trial results. Patients will be given time to read the PIS and have an opportunity to ask any questions they may have about participation in our study.

A consent form will be provided indicating that: the information given, orally and in writing, has been read and understood; participation is voluntary and can be withdrawn at any time without consequence. The consent form will also cover consent for access to medical records for ongoing data collection and follow-up.

After verifying that the PIS and consent form are understood, the person seeking consent will invite the patient to sign the consent form and will then add their own name and countersign it. A copy will be given to the patient, a copy placed in the patient’s medical notes, and the original kept in the Investigator Site File. If the patient is unable to physically sign the consent form (e.g. due to weakness, reduced dexterity), an independent witness (i.e. someone not involved in the trial) can sign on their behalf.

In the situation where a patient is approached in hospital but wishes to have more time to consider participation, they can request to be approached via the method detailed in 5.12.4.

#### Personal consultee opinion

It will usually not be possible to involve trial participants in the consenting process early on. Instead, consent will be obtained from patients once they have stabilised and are deemed to have capacity.

In the interim, once notified of the enrolment of a patient into our study, a delegated member of the site research team will approach the personal consultee (in person or via telephone) as soon as appropriate and practically possible to discuss the trial and seek their opinion as to the patient’s likely wishes and feelings regarding participating in the trial. This approach would take place as soon as reasonably possible, ideally within 24–48 h of randomisation, but could be most appropriate once the patient’s medical situation is no longer an emergency.

Where approached in person, the personal consultee will be provided with a personal consultee information sheet, containing all the information provided on the PIS, supplemented with information on why the personal consultee has been approached at this stage. Personal consultees will be given time to read the personal consultee information sheet and have an opportunity to ask any questions they may have about the patients’ participation in the study.

A personal consultee opinion form will be provided indicating that: the information given, orally and in writing, has been read and understood; the patients’ participation is voluntary and can be withdrawn at any time without consequence; and that, in the personal consultees’ opinion, the patient would not object to taking part in the trial.

After verifying that the personal consultee information sheet and opinion form are understood, the person seeking opinion will invite the personal consultee to sign the opinion form and will then add their own name and countersign it. A copy will be given to the personal consultee, a copy placed in the patient’s medical notes, and the original kept in the Investigator Site File.

If a personal consultee advises that, in their opinion, the patient would not choose to participate in the trial, then the trial treatment will be stopped (if ongoing) and the personal consultee asked whether, in their opinion, the patient would be willing to continue with ongoing data collection. Where a personal consultee is unable to visit the patient in hospital (e.g. due to infection control measures), this consultation may take place over the telephone. The consultation should be conducted by an experienced member of the site research team with knowledge of intensive care. The telephone consultation should be witnessed by another member of staff. The personal consultee information sheet may be sent to the personal consultee by email or by post. The outcome of the consultation will be documented and signed by the person seeking opinion on the personal consultee telephone opinion form, countersigned by the witness.

Upon patient recovery, the patient will be approached directly for informed deferred consent. The patient’s decision will be final and will supersede the personal consultee where there is disagreement.

#### Nominated consultee opinion

In the situation where the patient has died, a nominated consultee will be appointed. The nominated consultee may include an independent mental capacity advocate appointed by the NHS Hospital Trust or an independent doctor (i.e. not involved in the trial). The opinion of the nominated consultee will be sought in the same manner as for the personal consultee. A nominated consultee will also be approached in the rare situations where no personal consultee is available (or one is available, but does not wish to be consulted). Upon the patient’s recovery, the patient will be approached directly for informed deferred consent. The patient’s decision will be final and will supersede the nominated consultee, where there is disagreement.

#### Discharge prior to consent being confirmed

In the situation where the patient is discharged from hospital with mental capacity prior to confirming their consent decision, an experienced member of the site research team with knowledge of intensive care will attempt a phone call to the patient as soon as reasonably practical, such as within 2–3 working days following ultimate hospital discharge to inform them of their involvement in our study; provide information about the trial; and seek their consent. The telephone consultation should be witnessed by another member of staff. The patient information sheet may be sent to the patient by email or by post. The outcome of the telephone call will be documented and signed by the person seeking consent on the telephone consent form, countersigned by the witness. Prior to contacting the patient, the member of the local research team should check the participant’s electronic record to ensure there is no evidence that this is not appropriate, for example that their general practitioner or hospital has not issued a notification of death.

If there is no response to at least three telephone call attempts, or where no telephone number for the patient is documented, then the patient will be notified of their participation by post. The patient will be sent a patient notification letter, personalised by the most appropriate clinical/research team member, and a copy of the PIS, again following review of the patient’s electronic record for updates on their clinical condition. The letter will direct the patient to the PIS for detailed information on the trial and provide contact details for if the patient wishes to discuss the trial further or to opt out. In addition, the letter will confirm that the participant’s data will be included in the trial unless they notify the site research team otherwise.

Both methods described above will provide patients with the opportunity to opt out of ongoing data collection or follow-up questionnaires. A decision to opt out during the telephone call will be documented by the person seeking consent on the telephone consent form. For the postal approach, the patient can actively opt out by using the telephone contact details provided on the PIS, at any point during the trial.

If the participant is transferred to another hospital participating in the trial before the consent procedures are complete, then the local research team will contact the research team at the receiving hospital to hand over the consenting procedures.

#### Refusal or withdrawal of consent

If a patient declines informed deferred consent, or a consultee advises that they believe the patient would not choose to participate in the trial, and if a patient or their consultee (personal or nominated) withdraws consent/opinion at any time during the trial—this decision will be respected and will be abided by. All data up to the point of this decision will be retained in the trial, unless the patient or consultee requests otherwise.

For patients who decline informed deferred consent, or a consultee advises that they believe the patient would not choose to participate in the trial, but do not request removal of all data, the site will enter minimal pseudonymised data required for the primary outcome only. The justification for this is that as deceased patients cannot refuse consent to continue in the trial, excluding primary outcome data from the cohort of patients who survive to decline informed deferred consent would introduce substantial bias and impact upon the safety monitoring/reporting and, ultimately, the scientific validity of the trial and may prevent evidence of significant clinical benefit from being detected. No patient identifiable data will be recorded, and no further contact with the patient/consultee about the trial will be required.

### Study stopping rules

The study may be prematurely discontinued by the chief investigator (CI) or funder based on new safety information or for other reasons that come to light, including a lack of recruitment, that indicate the study should be discontinued.

### Safety

Adverse event (AE) reporting will follow Health Research Authority guidance on safety reporting/Medical Devices (MEDDEV) guidance (https://ec.europa.eu/docsroom/documents/16477/attachments/1/translations).

Severity and causality of each with use of the device as per the above guidance. Adverse events and device deficiencies will be recorded by the research or clinical team and reported directly to the site principal investigator using the adverse event reporting form available here as a supplementary document at the above link and available here: https://ec.europa.eu/docsroom/documents/16477/attachments/2/translations. AEs identified in Tables 3 and 5 will be recorded daily by the research team whilst the participant remains within the trial.

An event assessed as ‘severe’ or ‘life-threatening’ will be considered a serious adverse event (SAE) in this trial. Considering that all eligible patients are critically ill and at increased risk of experiencing multiple AEs due to the complexity and severity of their condition, unexpected adverse events are only recorded if they meet the criteria for an SAE and are considered to have occurred as a consequence of the trial itself (i.e. deemed to be ‘possibly’, ‘probably’, or ‘definitely’ related to the trial procedures). Any SAEs, or device deficiencies that would have led to an SAE had suitable action not been taken, intervention not been made, or circumstances been less fortunate, will be reported by the sponsor of the clinical investigation to the UK National Competent Authority, using the standard MEDDEV SAE reporting form and summary tabulation immediately, or at most within the timelines laid out in the above guidance.

Staff members involved in the care of patients enrolled in the study will have the ability to report any concerns regarding adverse device effects and device deficiencies locally to the CI or directly to the central trial group via email contacts displayed at each trial centre.

Any concerns raised by trial teams at each centre, or the Trial Management Group, may result in a pause in the trial data collection pending formal review. This could include the formation of a formal Data Monitoring Ethics Committee.

### Statistical analysis

We will test for a treatment effect using a linear mixed effects model with repositioning time as the response, fixed effects for site, sequence group assignment, and repositioning method, with random effects for individual to account for repeated measures. Analysis of the number of staff needed to carry out the repositioning procedures will take a similar approach, using a mixed effects cumulative logistic model to account for the discrete nature of the data. All tests will use the conventional 5% significance level.

No imputation or other statistical adjustments for missing data will be undertaken for these analyses. All recorded measurements of repositioning time and the number of staff needed within each proning session for the analysis, which means there is a very low chance that patients will be excluded from the analysis based on missing data. It will not be possible to tell if data on a repositioning exercise is fully missing. Whilst estimates of the treatment effect stratified by case complexity and BMI would be of interest, no sub-group analyses are planned as these would not be statistically reliable given the size of the trial.

This trial is focused on establishing efficacy in relation to the core repositioning outcome. Demonstrating efficacy is a necessary step before progressing to a larger trial that could robustly assess clinical outcomes, such as the prevention of pressure ulcers, which are relatively rare and require significantly larger sample sizes to detect with confidence. As such, this study is not powered to detect statistically significant effects in secondary outcomes, and formal hypothesis testing for these could be misleading. However, collecting and reporting detailed summary statistics will allow assessment of trends, variability, and effect sizes, which are essential for designing an appropriately powered future trial.

Summary statistics and distributional properties for the secondary outcomes will be produced and reported to inform any future studies focusing on patient outcomes and provider attitudes. The distributional properties of the continuous variables will be examined with histograms and boxplots. Participant characteristics and outcomes will be summarised using descriptive measures: mean (standard deviation) or median (interquartile range) for symmetric or skewed continuous variables, respectively; number (percent) for categorical variables. The findings from this study will provide essential data on usability, safety, and performance under clinical conditions, supporting the next stages of product development. These include refinement of the design, the definition of performance claims, and planning for regulatory approval.

The health economic evidence will be compiled to allow cost-effectiveness analysis of the BathMat technology relative to alternative technologies. Health economic measures will be reported in cost per QALY with mean and standard deviations, as with other secondary outcomes, to contribute towards the health economic argument for use of the device. In addition to standard health economic measures, bespoke information specific to the trial context will be collected. This tailored methodology will capture effects which cannot be captured by standard health economic measures. For example, the relationship between the number and frequency of repositioning (and associated costs), as well as the potential impact on complications, follow-up treatment, and subsequent litigation, cannot be fully captured using conventional methods alone.

## Data management

### Source data and documents

When a participant is enrolled, their information will be captured onto a participant recruitment form, which will allocate a unique participant identification number to the patient. Subsequent data collection will use this study number to identify the patient and record timing and clinical details onto the trial data collection form. Standardised data entry procedures will be followed across all sites to ensure consistency, with range and logic checks applied during entry to minimise errors. Key variables may be subject to independent verification to ensure data accuracy.

Once the patient has recovered from their critical illness, consent for their inclusion in the trial will be obtained by a suitable representative of the local hospital research team. Personal data will be stored on Excel spreadsheets saved on secure, password-protected servers within the RUH and on encrypted RUH laptops. Each trial site will have its own unique participant recruitment and trial data forms, with each research team able to access forms relevant to their participating site only. Clinical teams will have access only to data entry forms and will not be able to review patient data.

Any information that is analysed or transferred outside the clinical trial sites will be anonymised. Paper copies of any study documents, including consent forms and clinical letters with personal identifiable data, will be stored in a locked filing cabinet in an access-controlled area. Participant details will be anonymised in any publications that result from the study. Source data for this study will include certified scanned copies and/or paper copies of the consent form designed specifically for the study. An audit trail will be maintained for any changes to source data or electronic records, including time, date, and reason for the modification.

Patient notes will not be accessed by any member of staff outside of those involved in the direct care team until consent for this access has been obtained. Should the participant not survive their acute illness, only data collected for the primary outcome of the study will be used.

### Data handling and record keeping

Data will be collected and retained in accordance with the UK Data Protection Act 2018 and UK General Data Protection Regulation (GDPR). For this study, research data will be kept for at least 10 years. Personal data (e.g. name and address, or any data from which a participant might be identified) will not be kept for longer than is required for the purpose for which it has been acquired. Procedures will be in place to monitor data completeness and validity, and any discrepancies will be followed up with the local research team.

### Access to data

For monitoring purposes, the CI will allow persons responsible for the audit, representatives of the REC, and other regulatory authorities to have direct access to source data/documents. The trial manager (in collaboration with the CI) will manage access rights to the data set. Prospective new users must demonstrate compliance with legal, data protection, and ethical guidelines before any data are released.

Patient identifiable data (containing name, date of birth, patient identification number) will only be accessible by clinical members of the research team to allow further data collection pre-publication, if required. Pseudo-anonymised data will be shared with the non-clinical members of the research team named as study contacts in this document for the purposes of statistical, mechanical engineering, and health economic analysis. These data will identify participants by their study number only. Role-based access controls and password protection will be applied to all electronic datasets.

### Archiving

The RUH Bath NHS Foundation Trust is the data custodian for this study. All research data will be retained in a secure location during the conduct of the study and for at least 10 years after the end of the study, when all paper records will be destroyed by confidential means. An archiving plan will be developed for all study materials in accordance with RUH Standard Operating Procedures. Individual sites will archive their site files, consent forms, and related documents (paper and electronic) according to their hospital archiving policies.

### Access to the final study data set

Members of the Trial Management Group (TMG) will develop a data sharing policy that is consistent with the RUH Bath NHS Foundation Trust and University of Bath policies. It is anticipated that anonymised study data will be kept for future analysis and may be shared with other researchers, including those outside of the UK, European Union (EU), and European Economic Area (EEA), to enable international prospective meta-analyses.

The final study data set will be stored as restricted data on the RUH’s research data repository. Data will be made available to approved bona fide researchers only after their host institution has signed a data sharing agreement, which will be issued by the Research and Development (R&D) department and confirmed by the CI or appointed delegate. Details of how to request access will be available at the RUH R&D website.

### Monitoring of the final study dataset

The data collected for primary and secondary outcomes will be stored on NHS servers as a Microsoft form/Microsoft Excel spreadsheet. This will be backed up twice weekly to minimise any risk of data loss. Data entered will be reviewed by the CI or trial manager, and any concerns regarding data validation will be raised with local study teams for further assessment and consideration of any remedial actions required.

### Study management

The CI will take overall responsibility for managing the various components of the study, with the support of the trial manager(s) and will meet regularly (as required) with the leads for each component and PIs for each research site.

### Trial Management Group

The TMG will have responsibility for the day-to-day management of the study. The TMG will comprise the CI, all co-investigators, the trial manager, and other key study staff. The TMG will meet on a regular basis with a core working group of staff having weekly progress meetings.

### Trial Steering Committee (TSC)

The TSC will have oversight of the study and will meet every 3 months throughout the study to review progress and provide guidance on future plans. The Trial Steering Committee will be responsible for overall supervision on behalf of the sponsor and funder and will ensure that it is conducted in accordance with the relevant guidelines and regulations.

It will consist of the Chair (Dr Andy Georgiou), voting members (Prof. Tim Cook, Prof. Jerry Nolan, Prof. Mark Tooley, Lucy Dallyn, Prof. John Hailey, Prof. Veronica Hope-Hailey), and observers (Jane Carter, Stuart Haylock, Jerome Condry, Alexander Lunt), with additional observers attending as required at the request of the Chair.

### Patient and public involvement (PPI)

Before data collection, a PPI group has been recruited and convened to review the trial design, device, and consenting process. Their feedback has been used to update the device and protocols as required.

During data collection, leaflets and posters will be available to recruitment sites with information for patients, families, and staff, with details on how to get more information about the device and trial through our project website, and to contact the study team with questions. Anyone making contact will be offered the opportunity to receive monthly updates about the project. The PPI team leader recruited prior to this trial will also sit on regular steering group meetings, providing regular updates to the team on PPI input on the device.

### Funding organisation

This study (reference number NIHR206410) is funded by the NIHR.

## Monitoring, audit, and inspection

### Monitoring

The study will be overseen by the Trial Steering Group and the Trial Management Committee, with a central monitoring plan agreed in advance of trial commencement. Oversight will focus on enrolment progress, protocol adherence, and intervention fidelity. Recruitment rates will be reviewed on a regular basis using site enrolment logs and central screening logs. Any site not meeting expected enrolment targets will be supported through targeted engagement, additional training, and reclarification of eligibility or consent procedures as appropriate. Regular communication with sites will ensure that any local barriers to recruitment are identified early and addressed promptly to protect trial timelines.

To support consistent delivery of the intervention, the central research team will implement structured training for all personnel involved in trial delivery. This training will be accompanied by clear procedural documentation and checklists. Adherence to the intervention protocol will be assessed using routinely collected trial data. The research team will review these data regularly to assess consistency across sites, including confirmation that the intervention was delivered in accordance with the planned sequence and within appropriate timeframes. Any deviation from the expected protocol will be recorded and reported to the trial manager and chief investigator, and if systematic, investigated and addressed through direct engagement with site teams.

Auditing of the trial will be conducted in accordance with the agreed monitoring plan. The NIHR will oversee trial conduct through regular review of progress, documentation, and reported deviations. Audits or inspections by the Research Ethics Committee or other regulatory bodies will be undertaken if required. The process for audit is independent of the sponsor and investigators and will be managed through NIHR oversight structures, which include personnel with extensive experience in the conduct and evaluation of clinical trials. All documentation will be available for inspection as required.

### Notification of serious breaches to the protocol

A ‘serious breach’ is a breach which is likely to affect to a significant degree:

- The safety or physical or mental integrity of the subjects of the study
- The scientific value of the study

The CI and TMG will be notified immediately of any case where the above definition applies during the study conduct phase. This will include any clinical decision to remove the participant from the study or deviate from the above protocol other than by withdrawal of consent or change of opinion as no modifications are allowable within the protocol.

### Data monitoring plan

Data will be monitored for completeness at least weekly, and any issues with data completeness will be fed back to site research teams.

Further to ensure robust data is collected, each study site will have their first participant data monitored using a remote monitoring process, where sites will be asked to confirm via a self-audit that there is complete and accurate data held for this patient. Dependent on the outcome of this first remote monitoring, the following patient may be monitored, and this will continue until a site is able to report complete and accurate participant data. Once this is achieved, the site will complete this self-audit process approximately every 6 months or every 4th patient, whichever is sooner. This frequency will be reviewed by the chief investigator and trial manager throughout the study, and this document updated if changes are required.

If weekly data checks or monitoring processes highlight repeated issues or serious adverse events (SAEs) as defined in the protocol, then further monitoring may be triggered. This may be remote or in person by the trial manager at the study site, depending on the issue discovered.

**Ethical and regulatory considerations**

### Governance and legislation

This study will be conducted in accordance with:

- Data Protection Act 2018
- General Data Protection Regulation

For all amendments, the CI or delegate will confirm with the REC, RUH, and the research site that permissions are ongoing.

This study will take into consideration Good Clinical Practice (GCP). The key principles include providing public assurance that the rights, safety, and well-being of study participants are protected and that the clinical study data are credible.

### Research ethics committee review and reports

Ethics review of the protocol for the study and other study-related participant-facing documents, e.g. consent form, will be carried out by an independent NHS committee. Any amendments to these documents will be approved by the TMG before being submitted to the committee for approval prior to implementation.

All correspondence with the REC will be retained in the Trial Master File (TMF). Any reporting required to the REC will be in accordance with the conditions of approval.

Training will be carried out by certain staff members depending on their delegated responsibilities within the study; the level of training required will be determined according to the NIHR Delegation and Training Decision Aid. Informed consent to participate in the study will be sought and obtained based on GCP guidelines.

### Peer review

The proposal for this study has been peer-reviewed through the NIHR process, which includes independent expert and lay reviewers.

### Regulatory compliance

The study will comply with the necessary regulations and will not commence until a favourable REC opinion has been provided, and a letter of no objection for the Medicines and Healthcare Products Regulatory Agency (MHRA).

### Data quality

The quality of the study data will be monitored throughout the study by the central study team. If incomplete data sets are being submitted by the clinical team, the research team at participating sites will be informed to allow an action plan to be put in place.

### Indemnity

With no objection for the use of the device from the MHRA, indemnity for any adverse events arising from the use of the device will be provided by the hospital trust at each site and agreed in the research agreement prior to recruitment initiation.

## Dissemination policy

A plan for disseminating the study results will be developed by the TMG.

The main results of the study will be published in a high-impact peer-reviewed journal. Initial findings will be submitted to relevant national and international meetings. A lay summary of results will be provided to all participants who have consented to take part in the trial. An early-stage invention report will also be filed with any results collected pre-trial in an appropriate journal.

On completion of the study, a final report will be prepared for the funder, and once approved, made publicly available on their website.

## Trial status

Recruiting started in May 2025. The current protocol is version E of 24-01-2025. Patient recruitment is estimated to be completed around July 2026.

## Supplementary Information

Supplementary Material 1.

Supplementary Material 2.

Supplementary Material 3.

## Data Availability

Data collected for this study will be analysed and stored for 10 years from the completion of the study. When the study is completed, access to study data will be provided upon request to the principal investigators.
