# Supplementary material for: A randomised crossover trial of staff time with proned patients in the ICU using the ‘BathMat’
Source: Trials. 2025 Nov 13;26:503. doi: 10.1186/s13063-025-09221-x (PMC12613557; doi:10.1186/s13063-025-09221-x)
Supplement: Supplementary file 1 — Supplementary Material 1. [file 13063_2025_9221_MOESM1_ESM.docx]

Insert site specific logo here

IRAS ID: 333769

Centre Number:

Study Number:

Participant Identification Number for this trial:

**CONSENT FORM**

Title of Project: The ‘BathMat’ Trial: A trial of staff time with proned patients in the ICU using the ‘BathMat’

Name of Researcher:

Please initial box

1. I confirm that I have read the information sheet dated.................... (version...........) for the
   above study. I have had the opportunity to consider the information, ask questions and have
   had these answered satisfactorily.
2. I understand that my participation is voluntary and that I am free to withdraw at any time
   without giving any reason, without my medical care or legal rights being affected.
3. I understand that relevant sections of my medical notes and data collected during the study, may
   be looked at by individuals from the University of Bath, from regulatory authorities or
   from the NHS Trust, where it is relevant to my taking part in this research. I understand that any
   Information viewed by researchers at the University of Bath will only identify me by my trial number.
   I give permission for these individuals to have access to my records.
4. I understand that the information collected about me will be used to support
   other research in the future,and may be shared anonymously with other researchers.
5. I understand that the information held and maintained at this hospital and the Royal United Hospitals
   NHS Foundation Trust may be used to help contact me or provide information about my health
   status.
6. I agree to take part in the above study.

Name of Participant Date Signature

Name of Person Date Signature

seeking consent

Designation
