# Supplementary material for: A randomised crossover trial of staff time with proned patients in the ICU using the ‘BathMat’
Source: Trials. 2025 Nov 13;26:503. doi: 10.1186/s13063-025-09221-x (PMC12613557; doi:10.1186/s13063-025-09221-x)
Supplement: Supplementary file 2 — Supplementary Material 2. [file 13063_2025_9221_MOESM2_ESM.docx]

**Participant Information Sheet-SUMMARY**

**Research Study Title:** The BathMat Study: A trial of staff time with proned patients in the ICU using the ‘BathMat’

**Brief Summary**

In this research study we are trialing a new medical device designed to make caring for some of our most unwell patients safer. It is an inflatable mat, placed underneath patients while they are proned on Intensive Care. You have been given this leaflet because you have been included in our study while you have been unwell, and we would like to give you information about what has happened so far, and need your consent to use your information in our results.

In this research study we will use information from you] and your medical records. We will only use information that we need for the research study. We will let very few people know your name or contact details, and only if they really need it for this study.

Everyone involved in this study will keep your data safe and secure. We will also follow all privacy rules.

At the end of the study we will save some of the data in case we need to check it and for future research.

We will make sure no-one can work out who you are from the reports we write.

The information pack tells you more about this.

**Participant Information Sheet**

**Study Title:** The BathMat Study: A trial of staff time with proned patients in the ICU using the ‘BathMat’

IRAS ID: 333769

Principal Investigator: Local site leads details here

Chief Investigator: Dr Jerome Condry, Research Fellow in Intensive Care, Royal United Hospital, Royal United Hospitals NHS Foundation Trust, Combe Park, Bath, BA1 3NG

**Background and Purpose of Research**

We have designed an inflatable mat aimed at making managing proned patients on Intensive Care easier and safer for everyone involved. We call this device the “BathMat” You are receiving this information because you have been included in our study testing the device while you were on Intensive Care.

Proning is a way of helping people who are very sick and have trouble breathing. It involves lying patients on their front to get more oxygen into their body. When in this position, doctors need to turn the patients' head and move their arms every 2-4 hours. Doctors call this repositioning. It helps prevent sores as well as other injuries. This is currently performed by a team of 5+ staff and takes lots of time and resources. The process can also be dangerous because it requires a lot of movement which can hurt patients and staff.

To make repositioning easier and safer, a group of doctors and engineers have created a new device. It is like a cushion that goes under the patient and inflates. This allows staff to reposition patients without needing to slide the patient on the bed. This also reduces the number of staff needed and should lower the risk to patients and staff. A drawing of our device is pictured below.


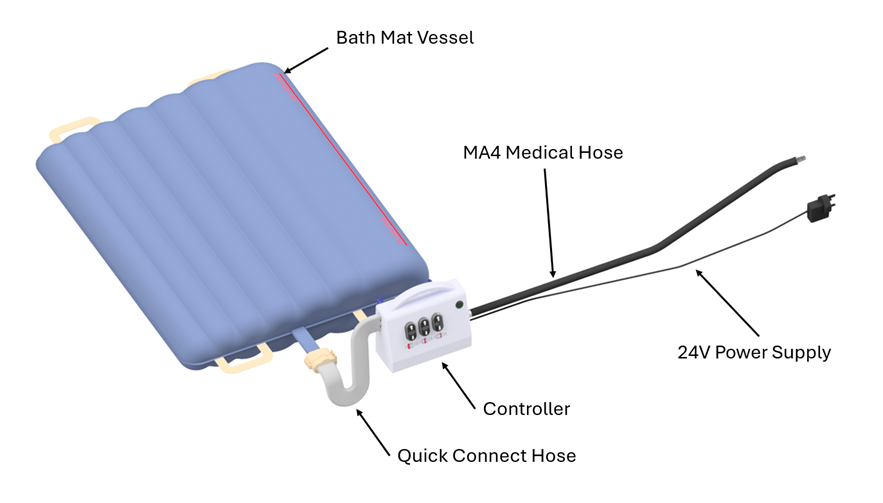


We are doing a study to see if our device works well compared with standard care in 30 patients. Patients in the trial will be repositioned using the device or usual care, and we will collect information on how well it performs, record any problems, and speak to staff to see what they think of the device.

Because our patients will be on Intensive Care as an emergency, we are only able to ask patients if they want to take part when they wake up. While you were asleep, we spoke to a member of your family or an independent doctor to see if they thought you would be happy to be involved in the study. We will ask patients if they want to take part and will collect feedback after they leave hospital.

To share the results with other doctors, we will write reports and give presentations. If successful, we will start making and using the device to help us care for our patients.

Everyone involved in this study will keep your data safe and secure. We will follow all privacy rules. At the end of the study, we will save some of the data in case we need to check it and to write scientific reports. We will make sure no-one can work out who you are from the reports we write.

The information that follows tells you more about this so you can decide if you would like your information to be included in our study

**Why are you being given this information?** While you have been a patient on Intensive Care, you have been included in a clinical trial supported by the National Institute for Health Research investigating the benefits of a new medical device designed to make caring for patients like you safer for everyone involved.

**What is the purpose of this study?** We are testing a newly designed inflatable pillow, placed under proned patients on Intensive Care, to allow them to be repositioned safely and efficiently. Our study is designed to test whether the device allows us to reposition patients safely and quickly with fewer delays to their care.

**Why have I been included?** You have been included because you were proned while on Intensive Care in an ICU which is taking part in the trial.

**What about my care changed because I was included?** On some days you were unwell staff may have used the BathMat to help move you. Staff then recorded the time it took, number of staff required, any delays to your care, and any complications you had due to proning.

**Why was my consent not sought prior to my inclusion in the study?** Because you were brought to Intensive Care as an Emergency, and we did not know if you would be proned until after you were asleep, we were not able to ask you before including you in the study.

**Who has been informed of my inclusion so far?** Once you were stable, a member of our research team approached a close friend or family member to ask them if they thought you would be happy to be included in the trial.

**What will happen to me if I decide to take part?** If you are happy to be included in the trial, we will use the data we have collected to use in our final results. We will also contact you again 1 to 2 months after you have been discharged from ICU for a brief interview to talk about the impact proning has had on your ongoing health. These interviews will take up to half an hour, and will only be necessary if our team has any questions after reviewing your notes. We will come to speak to you if you are still in hospital, or if not we will contact you via telephone.

**What are my options if I do not wish to be included?** If you do not wish to be included in the study, we will not make any further attempt to contact you. We will delete the information we have that allows us to identify you for the trial, and only use your data collected so far towards the main outcome of the trial. If you wish us to also delete the data we have collected so far, we will do this and none of your data will be used for our trial. You can request this when we discuss consent with you, or any time afterwards by contacting the research team using the details at the bottom of this document.

**Expenses and payment** We are unable to pay our participants for taking part. Each hospital will receive a small fee for each patient it recruits to cover the hospital’s costs in conducting this research.

**Will my involvement affect my ongoing care?** No. Ongoing involvement in this study will not affect the care you receive.

**What are the possible disadvantages of taking part?** Though we have tested the device using healthy volunteers, we do not know the benefits and risks of using the BathMat in patients on our Intensive Care. This is the reason we are performing the study. We expect using the device to improve the care received by patients using it. We do not expect there to be any disadvantages compared with the usual care you would receive, but are closely monitoring each patient for any evidence of increased risk.

**What are the potential benefits of taking part?** Involvement in the study while you were unwell may have benefited the care you received, but we will not have any evidence of this until the trial is completed. The benefits of you continuing to take part in the study are entirely altruistic. You may gain satisfaction in knowing that your contribution may help patients admitted to ICU in the future.

**What happens when the research study stops?** Once we have recruited a sufficient number of patients, we will assess the data we have collected to see if we can prove our device makes any difference to patient care. The results will be used in order to make a published report in a scientific journal.

**What will happen if I change my mind about being included in the study?** You are free to withdraw from the study at any time without giving a reason. This will not affect your future treatment in any way. Any data collected prior to your withdrawal may still be used in the final data analysis if you are happy for it to be used.

**What if there is a problem?** If you have a concern about any aspect of the study, we encourage you to speak to the researchers who will do their best to answer your questions. If your concern cannot be resolved by speaking to the researchers, you may also contact the Patient Advice and Liaison Service team at your recruiting hospital.

In the event that something does go wrong and you are harmed during the research and this is due to someone’s negligence you may have grounds for a legal action for compensation against your recruiting trust but you may have to pay your legal costs. The normal National Health Service complaints mechanism will still be available to you (as outlined above)

**Will my taking part in the study be kept confidential?** Yes. All participants will be issued with a unique identifier code which will be used to complete all further data entry into the study. A list matching participants with their unique identifier code will be kept in a locked filing cabinet along with an electronic version stored on an NHS password protected computer accessible only by authorised members of the clinical research team. All identifying information will be removed before data is written up for publication. Any information which leaves the clinical research team will have your name and address removed so that you cannot be recognised. Monitors and auditors from the NHS Research and Development offices may require access to participants’ personal data to verify or cross-check data as part of routine auditing.

Once this study has been completed, no-one will access your notes for the purposes of this study, but may use the anonymised data we have already collected to contribute towards future research on the device.

Data will be retained for a period of 10 years to allow any necessary cross checking. Anonymised data collected may be used in future studies.

**Do I have to give any samples or take any tests?** No. We are not collecting any samples or performing any laboratory testing as part of this study.

**Who is organising and funding this study?** The study is being organised by a team working at the Royal United Hospitals Bath NHS FT, working closely with a team at the University of Bath. It is funded by the National Institute of Health Research.

**Who has reviewed the research?** The research study has been independently reviewed as part of our application for funding from the National Institute of Health Research. All research in the NHS is looked at by an independent group of people, called a Research Ethics Committee, to protect your interests. This study has been reviewed and given favourable opinion by the Essex Ethics Committee and the MHRA.

**What will happen to the results of the study?** The fully anonymised results of this study will be presented at academic meetings nationally and internationally. They will be used to provide information to allow us to demonstrate the benefits of the BathMat for our patients and staff so we can continue to use it.

**How will we use information about you?** We will need to use information from you and from your medical records for this research project. This information will include your name, contact details, date of birth, and NHS number. People will use this information to do the research or to check your records to make sure that the research is being done properly.

People who do not need to know who you are will not be able to see your name or contact details. Your data will have a code number instead.

**What are your choices about how your information is used?** You can stop being part of the study at any time, without giving a reason, but we will keep information about you that we already have. You have the right to ask us to remove, change or delete data we hold about you for the purposes of the study. We might not always be able to do this if it means we cannot use your data to do the research. If so, we will tell you why we cannot do this. If you agree to take part in this study, you will have the option to take part in future research using your data saved from this study to help guide future development of our device. This data will be stored at the RUH and destroyed after 10 years.

**Where can you find out more about how your information is used?** You can find out more about how we use your information from: the HRA leaflet [[www.hra.nhs.uk/patientdataandresearch](https://www.hra.nhs.uk/patientdataandresearch)], by asking one of the research team, or by sending an email to [ruh-tr.FOIRequests@nhs.net].

**Further information and contact details** Thank you for taking time to read this Participant Information Sheet. If you have any questions, please direct them towards your local research team by email Insert local research email and contact details here If you wish to receive further information please contact our central team at TBC, or your local PALS team (Contactable at *Insert Local PALS contact here).
